# The role of food odor in invertebrate foraging

**DOI:** 10.1111/gbb.12793

**Published:** 2022-01-02

**Authors:** Nicolina Zjacic, Monika Scholz

**Affiliations:** ^1^ Max Planck Research Group Neural Information Flow Center of Advanced European Studies and Research (Caesar) Bonn Germany

**Keywords:** *C. elegans*, *D. melanogaster*, foraging, invertebrate, navigation, odor sensing

## Abstract

Foraging for food is an integral part of animal survival. In small insects and invertebrates, multisensory information and optimized locomotion strategies are used to effectively forage in patchy and complex environments. Here, the importance of olfactory cues for effective invertebrate foraging is discussed in detail. We review how odors are used by foragers to move toward a likely food source and the recent models that describe this sensory‐driven behavior. We argue that smell serves a second function by priming an organism for the efficient exploitation of food. By appraising food odors, invertebrates can establish preferences and better adapt to their ecological niches, thereby promoting survival. The smell of food pre‐prepares the gastrointestinal system and primes feeding motor programs for more effective ingestion as well. Optimizing resource utilization affects longevity and reproduction as a result, leading to drastic changes in survival. We propose that models of foraging behavior should include odor priming, and illustrate this with a simple toy model based on the marginal value theorem. Lastly, we discuss the novel techniques and assays in invertebrate research that could investigate the interactions between odor sensing and food intake. Overall, the sense of smell is indispensable for efficient foraging and influences not only locomotion, but also organismal physiology, which should be reflected in behavioral modeling.

## Graphical abstract

Foraging for food is an integral part of animal survival and odor‐guided navigation plays an essential role in finding food. We review how odors are used by foragers to move toward a likely food source and the recent models that describe this sensory‐driven behavior. We argue that smell serves a second function by priming an organism for the efficient exploitation of food.

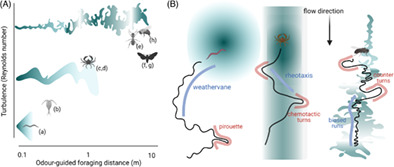

## 1 INTRODUCTION

Finding food is essential for animal survival. Thus, a diverse and adapted set of behaviors have evolved to allow animals to effectively forage in their niche environment. This adaptation to the environment, including its risks and its available resources, applies to invertebrates as well. This includes naive hawkmoths preferring the smell of flower tubes with an optimal length for their proboscis and feeding while in flight to avoid ambush predators.^1^ Female mosquito approaches unsuspecting hosts by first identifying plumes of exhaled CO_2_, then honing in on human skin odorants and visual cues.^2^ Ants track the sun and count their steps during foraging expeditions to find their way home again^3^, ^4^ and a species of hermit crab can forage across terrestrial, marine and freshwater environments,^5^ showing remarkable diversity.

Broadly, foraging involves a balance between exploration and exploitation, where exploration is the search for profitable food sources and exploitation is the localized search and feeding in a known food patch.^6^ Despite the variety of foraging strategies spanning diverse habitats, feeding apparatus and mobilities, the basic principles of foraging behavior between invertebrates can be well‐fitted by the same class of models.^7^ A key similarity is for example state changes between a fast exploration mode (roaming) and a slow, local exploitation mode (dwelling). This strategy is effective at maximizing food encounters and is present in both *Caenorhabditis elegans* as well as in *Drosophila melanogaster*.^8^, ^9^, ^10^

While multiple cues are integrated when navigating toward food, a key sense used in navigation across phyla is the sense of smell. Invertebrates from the crawling worm to the flying fruit fly all rely on odors to locate their next meal over long distances^11^, ^12^, ^13^, ^14^ and the mechanisms of invertebrate olfaction in general are well understood,^15^ especially in mosquitos and flies. Recent work has developed multiscale connections spanning from the structure of the key odor receptor family Orco,^16^ to behavioral and neural data linking navigation and the coding of odor stimuli^17^ and finally to the connectome and projections of the antennal lobe, which is the key brain area for sensory integration in insects.^18^, ^19^ In contrast, how odor affects food intake among invertebrates is vastly understudied. Anticipatory feeding behaviors, such as motor^20^, ^21^, ^22^ and digestive priming,^23^, ^24^, ^25^, ^26^ prepare the animal for rapid ingestion and reduce the time spent exposed to predators,^22^, ^27^, ^28^ for example. One can therefore argue that odor cues serve a second and equally vital function during foraging by preparing the animal for efficient food exploitation (odor priming), which deserves greater attention in the fields of invertebrate foraging and olfaction.

To gain a complete understanding of both exploration and exploitation in foraging requires simultaneous measurements of animal motion, food intake and odor concentration. Although it would be desirable to perform such measurements in natural environments with realistic odors, currently studies are only fully tractable using laboratory animals. Nevertheless, invertebrate model organisms are particularly suited for investigating the role of smell in exploitation because of their small size, early maturation, large brood sizes and the plethora of genetic tools and databases that are already established.^29^, ^30^, ^31^, ^32^ Food intake can be well monitored and accurate assays can be designed to mimic increasingly more realistic odors and resource distributions,^33^, ^34^, ^35^ hence invertebrate models can help fill the gaps in our understanding of foraging behavior.

In this review, we cover the role of odors in invertebrate foraging, with a focus on data from model invertebrates like *D. melanogaster* and *C. elegans*. We first briefly summarize how odor is used as a cue in exploration before presenting evidence for odor's role in exploitation. Moreover, we will discuss current models of foraging behavior and how they can better reflect organismal energy balance by explicitly accounting for odor priming. Lastly, we suggest experiments based on recent advances in technologies and assays quantifying invertebrate behavior in order to better understand the role of odor sensation in both the exploration and exploitation phases of foraging.

## 2 EXPLORATION—ODOR AS A CUE

### 2.1 Smell is the key sensory modality for food detection

While invertebrates can access multiple sensory modalities, the odor remains one of the most important cues for foraging.^11^, ^12^, ^13^, ^36^ Unsurprisingly, in *Drosophila* larvae and *C. elegans*, sensing food odors confers a fitness advantage by allowing animals to detect scattered food sources or through odor‐dependent alterations in the organismal metabolism that result in prolonged lifespans.^37^ Furthermore, odor‐sensory cues are vital for the efficient localization of food sources in specific niches. When naive hawkmoths from two species of the same subfamily were presented with odor and a visual stimulus in a wind tunnel, nocturnal *Deilephila elpenor* placed more importance on the odor, while the diurnal *Macroglossum stellatarum* strongly preferred the visual stimulus.^38^ This is in line with the hawk moth subfamily's nocturnal ancestry, where their keen sense of smell allows them to locate food in the dark when visual stimuli such as flower shape and color can be less reliably distinguished. Consequently, hawkmoths are set apart from visual, light‐dependent foragers like *M. stellatarum* by partitioning themselves temporally into a different niche, thereby reducing competition and improving resource acquisition.

Odors can also travel farther than sightlines allow in dense terrain, and odor plumes can stretch up to tens of meters.^39^ In particular, long‐range foragers such as bumble bees, moths and certain marine invertebrates rely on odor cues to guide their movements.^39^, ^40^, ^41^, ^42^, ^43^ For example, the bumblebee relies solely on odor rather than incorporating visual cues when locating small flowers. Visual cues only replace odor in cases of large flowers with a low probability of odor encounter.^44^ Evidence suggests female mosquitos begin host localization by first scenting fluctuating CO_2_ levels downstream of a human as far as 10 m away. After following the CO_2_ plume within a 1 or 2‐m proximity of the target, they may then leave the plume and only rely only on human skin odorants and visual cues for close approach and landing.^2^ Although army ants are known to have exceptionally poor vision, as is the case with *Eciton hamatum*, they are still able to locate the few species of ants that make up their specialist diets by detecting and discriminating among the many odors present in their neotropical habitat.^45^ Smell is therefore a key mid‐ to long‐range sense in diverse habitats.

### 2.2 Common principles of odor‐guided navigation at different scales

**FIGURE 1:**
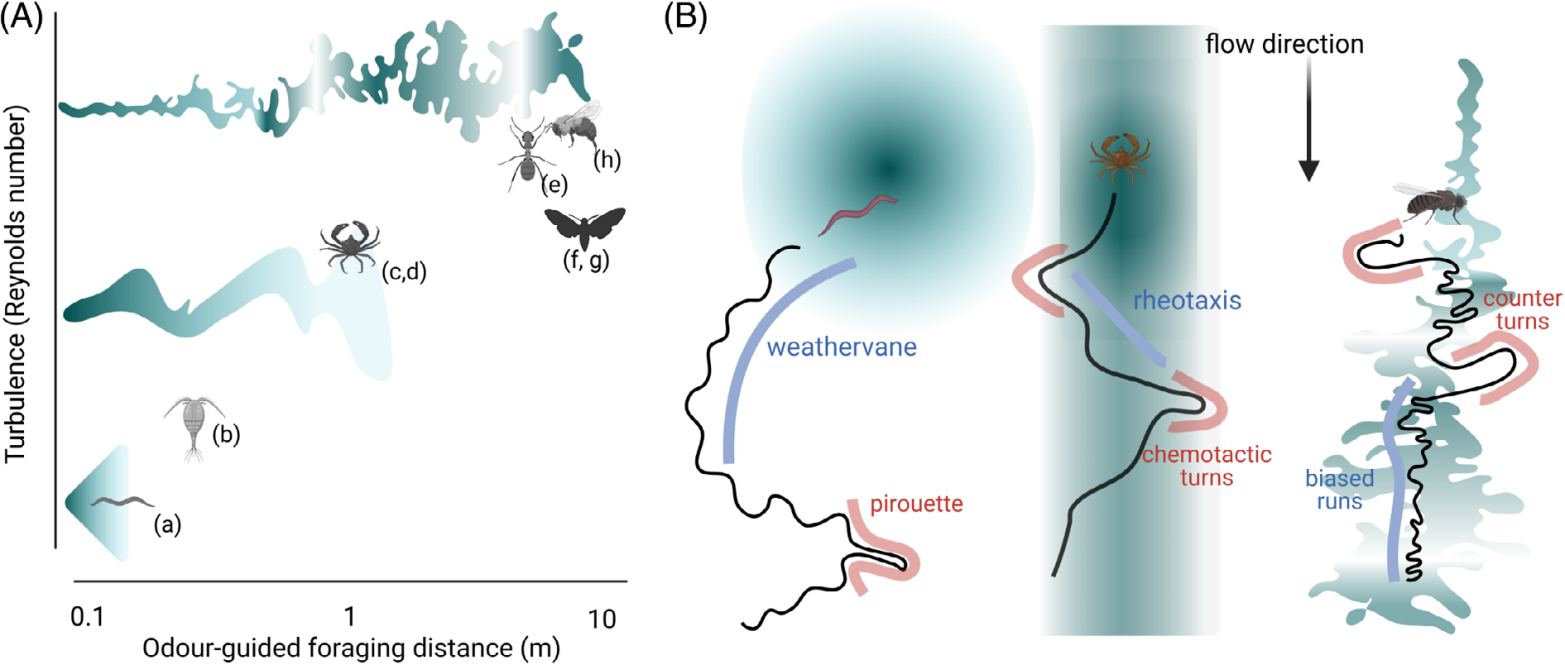
Typical odor plumes and navigational strategies for invertebrates. (A) Typical plumes and odor‐guided foraging lengths encountered by roundworms (a),^46^, ^47^ copepods (b),^49^ blue crabs (c)^42^ or green crabs (d),^43^ desert ants (e),^51^, ^52^ gypsy or hawk moths (f, g)^41^ and bumble bees (h).^39^, ^40^ Odors transition from smooth gradients to turbulent odor plumes with increasing travel distances. (B) Navigational strategies of three species encountering odor plumes of different types. *C. elegans*,^46^, ^47^, ^48^ blue crabs^50^ and walking *D. melanogaster*.^53^ Irrespective of the strategy followed, navigation can be divided in runs (light blue) and turns (pink). Odor concentration is shown in dark green

Effective navigation in search of food depends on the specifics of the environment, and particularly what form the odor cue is expected to take. In environments with laminar flow, odorants form stable, long gradients that allow for smooth gradient tracking such as chemotaxis (following a chemical signal).^46^, ^47^, ^48^, ^49^ Moderate wind or water flows result in longer odorant tubules that still show well‐defined gradients, but must be integrated into the animal's tracking through rheo‐ or anemotaxis (movement following the water‐ or wind direction^42^, ^43^, ^50^). With larger wind or water flow speeds, odor plumes become turbulent, therefore requiring complex and rapidly adapting search strategies to effectively navigate (Figure 1A).^39^, ^40^, ^41^, ^51^, ^52^ Locomotion plays an integral part in how animals experience these odor plumes. Depending on the animals' size and speed of locomotion, they will sense vastly different time‐varying odor stimuli as they move relative to the odor. Consequently, the animal tracks from these three navigational regimes will show distinct statistics of behavioral parameters such as run length, turn direction and frequency and directional bias (Figure 1B).

For example, the roundworm *C. elegans* in the laboratory mostly experiences laminar flow or static environments with smooth gradients. When *C. elegans* navigates to a food patch, it will use random reorientations called pirouettes and smooth curved trajectories as it reorients toward the highest odor concentration (Figure 1B), reacting to the smooth increases in odor concentration as it approaches the food.^46^, ^47^, ^48^ In contrast, blue crabs encounter smooth odors on a background of active flow, thus leading to navigation upstream along “tubules,” with chemotactic (chemical‐induced) turns at the edge of the odor column.^42^, ^50^ Surprisingly, a similar “counter‐turning” behavior at the edges of an odor stripe has also been shown in worms embedded in a flow environment, despite crabs being orders of magnitude larger in size and typically experiencing much larger flow rates.^54^

Airborne insects like moths or *D. melanogaster* experience a very different environment, which is dominated by air flow that will disrupt any smooth gradients and result in turbulent plumes that are neither continuous, nor form a stable, smooth gradient of chemicals (Figure 1A). The navigational strategy of walking flies is thus composed of runs biased toward the (expected) odor source, and counter‐turns when the plume is lost^55^ (Figure 1B). Interestingly, a similar strategy is employed when aquatic arthropods encounter turbulent plumes.^56^ Without smooth odorant gradients simple strategies that work in laminar environments fail, for example, comparing the current and preceding odor concentrations to determine which direction is closer to the source. Given their greater speed, the sensory experience is even more complex for flying insects, as their own wingbeat can cause changes in the odor plumes.^57^ Such environments require adapted sensory strategies, which often integrate both the odors, as well as the wind speed and the animal's own wingbeat frequency to determine an optimal flight direction.^58^, ^59^, ^60^ Further investigation is needed for predatory feeding, where both the food source (prey) and the predator are moving. In this case, the landscape of odors and locomotion is even more complex, which is beyond the scope of this review.

It is surprising that similarities in odor tracking strategies were identified despite the different environmental characteristics, scale and type of locomotion.^61^ At its core, any navigational strategy consists of runs in the estimated direction of the odor source, and re‐orientations when the odor is lost or reduced. Extracting statistics from animal tracks during odor tracking and comparing these to stochastic processes such as biased random walks, continuous‐time random walks or Lévy flights has elucidated the mechanisms involved in generating these trajectories.^48^, ^62^ In turn, these stochastic models make predictions about the required neuronal activity underlying these navigation behaviors.^63^, ^64^ Overall, odor tracking in navigation has been extensively studied across species and is thus relatively well understood.^7^

## 3 EXPLOITATION—ODOR AS A PRIMER

### 3.1 Odors help appraise the value of food sources

**FIGURE 2:**
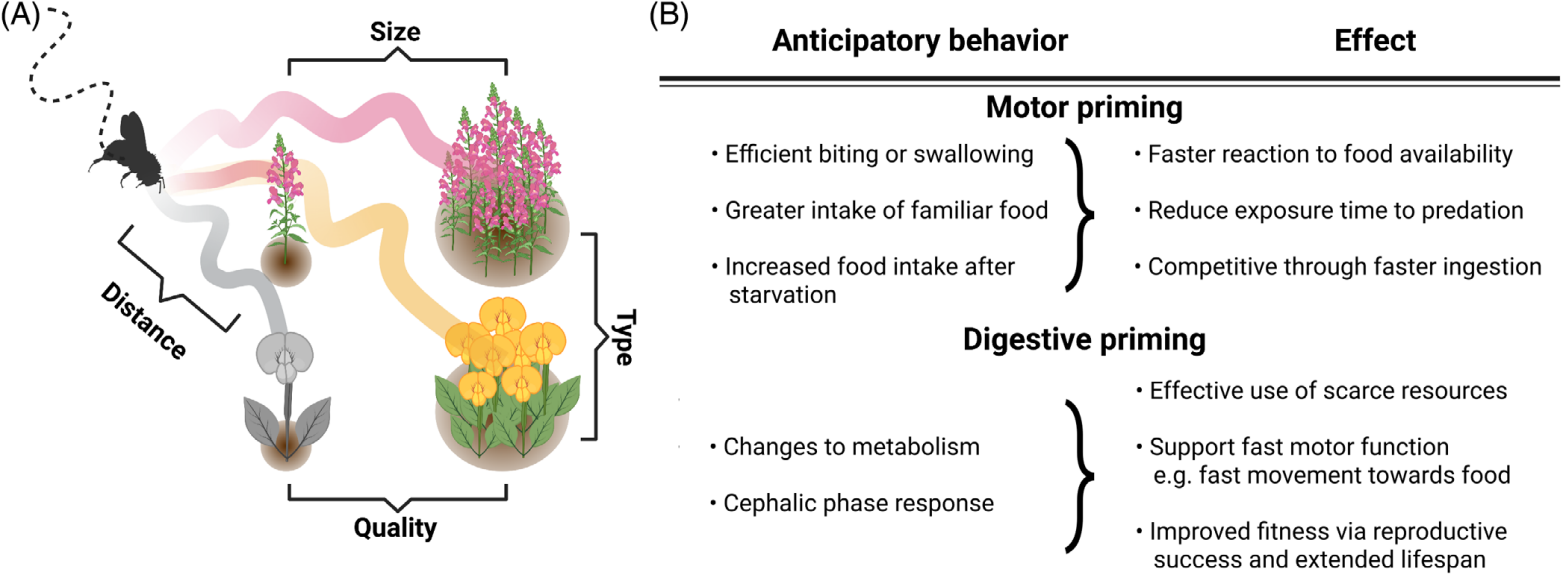
Advantages of odor sensation during exploitation. (A) Besides determining distance to food, odor plumes allow for distinctions between food type, quality and size and allow animals to navigate based on nutritional needs and other internal drivers. (B) Anticipatory feeding behaviors induced by food odor, including the motor‐ and digestive priming, allow for improved animal survival, fitness and reproduction through efficient food capture and ingestion

Odor not only indicates the direction toward a food source but also its quality and type, leading to the more effective exploitation of high‐value food or food filling a nutritional gap, when the animal is provided with a choice (Figure 2A). Besides fulfilling energy requirements, food choices additionally satisfy demands for specific nutrients.^65^, ^66^ For example, terrestrial gastropods selectively choose plant species and fresher leaves to feed on through olfaction.^67^ *Drosophila* adapt foraging choices to amino‐acid needs based on odors, such as females replenishing their reserves after mating by taking more sips of amino acid‐rich yeast compared with sucrose, while virgin flies are uninterested in yeast.^68^ More than 70 years ago, Lindauer showed that the addition of scent to a known unscented food source led to an initial decrease in honeybee waggle dances compared with controls, where the number of waggle dances was used as a proxy for food attractiveness.^69^ Ants will interrupt feeding if the scent of the food contradicts their prior experiences, even if the energy value of the food, as perceived by its sweetness, is exactly the same.^70^ *Drosophila* will prioritize food odor over the innately aversive odor CO_2_ when feeding on fermented fruit.^14^ Odor is, therefore, an integral part of food evaluation across multiple species, even to the extent of dominating multisensory inputs and spurring counter‐intuitive behavior.

Because of its prominence and tractability as a genetic model organism, olfaction and food sensing has been extensively studied in *C. elegans*. In the worm, both the neural circuits as well as the mechanisms of odor‐guided food selection are well understood. Worms display preferences for certain bacterial food sources over others^28^, ^71^, ^72^ and identify them based on their attractive odorants.^73^ When provided with bacteria found in their natural habitat, worms chose food with higher nutritional value, which then led to an extended lifespan.^28^, ^71^ Attractive smells further facilitate *C. elegans* feeding behavior in the presence of food via increased pharyngeal pumping, while repellent smells suppress it.^74^ In worms, the neurons primarily responsible for food sensing have been identified, as well as the navigational circuits that are active during foraging.^9^, ^75^ A neural “flip‐flop” circuit has been proposed linking odor‐sensory neurons AWC and AWB, as well as the neuropeptides they release, to the recognition and subsequent generation of food odor preference.^76^ When contradictory odors are encountered, this flip‐flop motif performs a nonlinear computation of the sensory inputs that leads to stable behaviors in noisy environments where multiple conflicting or fluctuating odors are present. Moreover, AWC is part of the circuit that drives local food‐searching behavior,^9^, ^77^, ^78^, ^79^ thus providing the neural circuit that connects odor sensation and the corresponding changes in locomotor behavior. *C. elegans* is therefore a highly useful model organism for studying the neural circuits linking odor and food exploitation.

Internal states such as hunger, mating drive or sleep drive similarly alter the perception of food value.^68^, ^80^, ^81^, ^82^ Internal states are often set by neuromodulators and neuropeptides, which signal gross changes in the balance between behavioral and sensory priorities.^83^, ^84^ For example, increased value can be allocated to odor sensation during times of hunger.^85^, ^86^ When placed on a single fly treadmill, hungry *Drosophila* will doggedly pursue a food odor even when no reward is forthcoming, meaning the promise of food driven by smell is enough to outweigh negative experiences.^87^ Furthermore, hungry *Drosophila* will pick up attractive, low odor concentrations of vinegar more acutely while reducing the neural activation caused by aversive high vinegar concentrations.^88^ Similarly, when *C. elegans* are starved they risk death to obtain a meal based on the food smell emanating across a dangerous desiccating hyperosmotic barrier.^89^ Odor sensation can thus affect food intake by altering the valuation of the food depending on current and dynamically changing organismal priorities.

### 3.2 Anticipatory digestive and motor behaviors lead to efficient feeding

Changes in metabolism as a result of food odor detection can cause more efficient uses of resources by priming the body for digestion (Figure 2B). Efficient digestion leads to beneficial effects such as greater longevity and fertility, thereby positively impacting the animal's fitness.^20^, ^22^, ^90^ However, in dietary‐restricted worms and flies, ablating odor‐sensory neurons leads to an increase in life‐span,^91^, ^92^ which in worms is mediated by an octopamine signal to the gut.^93^ While it is possible that this odor‐mediated change in lifespan reflects a necessary tradeoff between earlier reproduction when conditions are favorable, and faster aging,^94^ the behavioral and ecological function of odor‐mediated inhibition of longevity for the survival of the animals has not yet been established.

The cephalic phase response, or the preparation of the gastrointestinal tract for optimal food processing, has been described in rodents^95^, ^96^, ^97^ and humans, although the evidence from human trials is conflicting and debated.^98^, ^99^, ^100^ In model invertebrates, odors have been found to change lipid catabolism in peripheral fat storage tissues,^20^, ^22^, ^90^, ^91^ proteostasis^21^ and reproduction rates via germline proliferation.^94^ In *C. elegans* for example, the activation of odor‐sensory neuron AWC by the food‐related odor 2‐butanone causes a cascade of metabolic reprogramming of fat‐related pathways, leading to more efficient exploitation of energy sources without directly changing feeding behavior.^20^ Moreover, odor‐regulated microRNAs inhibit AWC, stimulating proteostasis and prolonging longevity.^21^ The odor‐sensory neuron AWB is responsible for detecting the presence of preferred dietary odors and adjusting germline proliferation accordingly, favoring increased reproduction rates and an early onset of reproductive aging to counteract increasing germline mutation rates with age.^94^ Other species, such as scavenging deep‐sea amphipods, anticipate food intake by increasing their initial oxygen consumption when exposed to bait odor. The amphipods then switch from an energy‐conserving state to an active one, allowing them to migrate quickly to the meal to exploit it and leave quickly to avoid predators.^22^ Digestive priming thus links odor sensing to exploitation by directly affecting and adapting metabolism to the available food sources (Figure 2B).

The second mechanism connecting odor sensation to increased food intake primes the animal by directly affecting the motor programs controlling biting or swallowing (Figure 2B). In *C. elegans*, the presence of attractive odors directly affects the feeding rate via the sensory flip‐flop circuit described above^74^ and a similar effect can be observed in *Drosophila* larvae.^101^ Starved *C. elegans* upregulate their feeding rate even in the absence of food, possibly to enhance ingestion when food becomes available again.^27^ This upregulation might allow the animal to immediately detect even small food sources and exploit them without delay, as worms also employ feeding as a way of sampling their surroundings.^102^ By preemptively increasing the rate of food intake animals can reduce the time spent in the potentially dangerous and vulnerable state of feeding. Beyond these examples in model organisms, few experimental studies have shown direct evidence of motor priming, likely because of the difficulty in detecting food intake in unrestrained animals. Yet, motor priming will directly affect measured foraging parameters such as time spent in a food patch and will therefore need to be considered. Overall, olfaction can additionally improve food intake and resource utilization by inducing digestive or motor preparation.

## 4 MODELS OF FORAGING BEHAVIOR IN DIFFERENT DISCIPLINES

**FIGURE 3:**
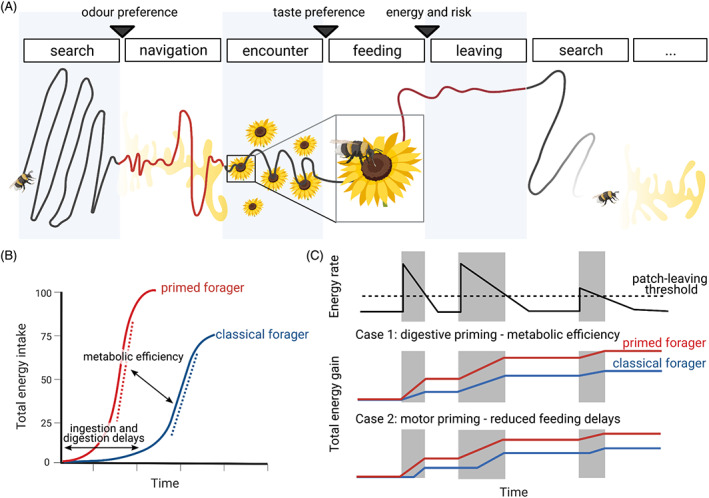
Foraging decisions are altered when a forager is primed by odor sensation. (A) Foraging encompasses multiple sub‐behaviors of which a forager can perform all or a subset. The animal starts with a random search pattern, and once an odor is encountered, odor navigation begins. Upon food encounter, feeding begins. Eventually, the forager leaves the current food patch. There are many decisions involved (black arrowheads): choice of a patch, taste and choice of leaving. (B) Anticipatory behaviors can alter energy intake, for example by shortening ingestion and digestion delays or by allowing a higher metabolic efficiency. (C) Possible effects of odor priming on a marginal value theory forager: The forager leaves a patch when the food intake drops below the average expected rate (top, dashed line is the average expected rate, gray is time in a patch). Given the same time spent in a food patch, both efficient digestion (middle) and motor priming (bottom) lead to more energy intake compared with an unprimed classical forager, either by faster metabolism or reducing ingestion delays, respectively

As a complex behavior, foraging encompasses many behavioral aspects that can be modeled, ranging from statistical models underlying the process of navigation, to inference of internal value models and the neural basis of decision‐making. A typical foraging sequence comprises five distinct behavioral phases: random search (without cues), cued navigation toward a food source, encounter, feeding and leaving (Figure 3A). Here, we focus on models that aim to implicitly or explicitly codify the animal decisions that affect the timing and duration of foraging phases. At most transitions between foraging phases, animals can decide to enter the next phase, continue the current phase or repeat the previous phase (Figure 3A, black arrowheads). Thus, most foraging models fundamentally try to model animal decisions: habitat choice, patch choice, choice of diet and patch leaving are all decisions the foraging animal makes repeatedly.^103^, ^104^ Similarly, food choice, feeding duration in a patch and patch leaving are three decisions that have been tested against data for many foraging species.^105^

In the classical paper by Charnov,^106^ the now well‐known marginal value theorem (MVT) was introduced. Economical in nature, the theorem connects the travel time between patches of food with the optimal time to leave a patch, given that the forager aims to maximize the rate of food intake relative to the energy loss because of motion, that is, its energy balance. The theorem's value lies in clear predictions for measurable foraging parameters such as the optimal patch residence time. However, the original model has been criticized for lack of experimental support since data showed a consistent bias toward the forager remaining in the patch longer than predicted by the MVT.^105^ Subsequent modifications, therefore, included arbitrary food distributions,^107^ time‐limitations for foraging because of other behaviors^108^ and underlying behavioral states.^105^

A different class of models from cognitive neuroscience use decision theory to explicitly describe the process of deciding, rather than its optimal economical outcome.^105^ While MVT assumes the forager has perfect knowledge of the available patches, decision theory models assume the forager has to decide based on unknown rewards, which it needs to measure during exploration of its environment.^109^ For example, an evidence‐integrating drift–diffusion model has been used to predict feeding decisions in *C. elegans* when the concentration of food is uncertain.^102^

More complex hierarchical decision models have extended these basic choice models to integrate internal states such as hunger, mating drive or satiety and motivational states.^110^

Foraging can also be modeled by considering the information that is available to the animal for its decision. These approaches explicitly account for and consider how animals could optimally collect information from fluctuating or noisy cues. The proposed strategy for navigation using optimal information gathering was termed “infotaxis.”^111^ A noisy cue, such as a scent in a turbulent flow, could lead an animal to a food source. Infotaxis considers that the animal can either navigate toward the likely odor source or gather more information, depending on how accurate its current estimate of the source location is. The elegant algorithm is tractable for simple biological implementation. To date, however, there is limited experimental evidence for infotaxis, in part because of the difficulty in measuring information acquisition and processing in foraging animals. A study in *C. elegans* has shown some evidence that worms might implement infotaxis after being removed from a food source.^78^ For patch leaving, a model has now integrated noisy information about the environment and food rewards with evidence accumulation from decision theory.^112^ To date, models describing all aspects of foraging from search to patch leaving at a level of detail that includes cues, evidence and information, remain elusive.

## 5 INTEGRATING SMELL INTO MODELS OF FORAGING AND FUTURE EXPERIMENTAL DIRECTIONS

Current foraging models account for odor cues when modeling exploration, or the navigation toward a food source. We suggest integrating anticipatory behaviors into existing foraging frameworks because of their impact on the dynamics of energy intake and changes to organismal risk assessment. To add the effects of priming into foraging models requires only small changes to existing models. We will here discuss how this adaptation could be achieved for the MVT model, as it is easy to illustrate how the effects of odor priming might affect the energy balance of the animal, which MVT aims to maximize.

Accounting for smell in the valuation of a food patch would mean adding an additional term to the cost function that describes food preference because of odor. Such preferences have been measured successfully for a large number of volatile and soluble compounds in binary or multi‐choice assays.^113^, ^114^, ^115^ Therefore, adding this aspect would not necessarily require free parameters, but rather adding a term that is, sign and strength are already established (Table 1).

**TABLE 1:** Food‐related odors and their behavioral effects on invertebrate animals

Species	Odor	Natural source	Behavioral effect	Reference
*C. elegans*	Isoamylalcohol (IAA)	Bacteria^73^	Increase in pirouettes	116
	Diacetyl (DIA)	Rotten fruit, lactic acid bacteria^117^	Attraction toward source smell	117
	2‐Butanone	Pathogenic bacterium *Serratia marcescens* ^73^	Attraction toward smell source	73
*D. melanogaster larvae*	Ethyl butyrate	Fruit, for example, physalis, cactus fig, honeymelon^115^	Attraction toward smell source	115
	Ethyl acetate	Fruit, for example, passionfruit^115^	Attraction toward smell source	115
	Methyl butyrate	Byproduct of microorganisms in soil and rotten vegetation^118^	Increased in pirouettes	119
*D. melanogaster* adults	Mthyl acetate	Fruit, for example, passionfruit^115^	Increase in walking speed	120
	Benzaldehyde	Almonds, fruits^121^	downwind movement	120
	2‐Phenylethanol	Fruit, for example, red currant^115^	Strong attraction toward source smell	115
*Aedes aegypti* (blood‐feeding females)	L‐lactic acid	Human skin odorant^122^	Attraction toward smell source in combination with CO_2_	122
	Eugenol	Clove, cinnamon, pepper^123^	Leaving smell source	124
	CO_2_	Air	Upwind reorientation	122

*Note*: Behavioral effects are frequently concentration‐dependent and might even invert at very high concentrations.

Similarly, adding the effects of anticipatory feeding behaviors such as digestive and motor priming would mean adding two terms: altering the term accounting for the expected nutritional benefit, and one accounting for delays in ingestion of unfamiliar food or lacking priming. Anticipatory behavior will also be important in the limit of small patches. If the forager is already primed to ingest, stopping at a smaller patch will be beneficial (Figure 3B,C). Examples of the possible effects of ingestion, digestion and motor priming on energy intake on the MVT are shown in Figure 3C. These are meant to be interpreted as hypotheses, and actual effects will depend on the exact foraging model and environmental conditions.^112^ Further aspects that could be explored in these models are the changes to risk assessment (Figure 3A) and patch residency because of faster food intake and faster digestion.

Measuring predictions of such foraging models requires simultaneous measurements or at least simultaneous inference of food intake, locomotion and odor inputs. The challenges of this task differ between field experiments and laboratory setups. The natural landscape, wind and water flows that shape the odor plumes encountered by animals in their natural habitats can hardly be reconstituted in a laboratory environment (Figure 1) and despite various available techniques for tracking animals ranging from satellite images,^125^ radar^126^, ^127^ or radio telemetry,^126^ these approaches lack the resolution to observe feeding behavior and are unsuitable for very small animals.^128^ In aquatic environments, ultrasonic acoustical tags are an option, but are relatively expensive and require injection of tags into animals.^129^ Because of weight, many nonoptical methods like radio frequency identification requiring tags are not feasible for small insects and invertebrates.^130^, ^131^ Even in larger animals, chipping can hinder their natural behavior, and with multiple meters of resolution wave‐based detection systems like radar are unsuitable to track fine‐grained animal motion. The most suitable approach to detect and track small animals are therefore optical measurements using camera systems.

The substantial decrease in prices for camera chips and the ubiquitous availability of consumer‐grade video cameras such as GoPros has vastly simplified the imaging of animal behavior.^132^, ^133^, ^134^ The remaining challenge is to automatically extract animal location and ideally posture from these videos for analysis. To tackle this, software tools that are broadly available such as DeepLabCut, TRex or SLEAP provide intuitive interfaces for training and analyzing image data in different contexts.^135^, ^136^, ^137^ While it is much easier to analyze laboratory data with simple backgrounds, good lighting and often multiple cameras that allow tracking even when parts of the animal are obstructed, these methods can also perform well on field data.^138^, ^139^ Recent work using deep learning with frame‐to‐frame predictive priors has made camera‐based tracking more successful, in certain cases even following animals while completely obstructed to a human observer.^140^ Video tracking and deep learning‐based analyses have thus revolutionized the field of animal tracking.^132^, ^133^, ^134^

When considering the complexity of the sensory landscapes experienced by animals during navigation, lab experiments with model animals retain their advantage. Odor inputs can be inferred, visualized or modeled,^141^, ^142^, ^143^, ^144^ but because of the spatio‐temporal dynamics of turbulent flow and the fact that the presence of the animals themselves alters the odor plume, these approaches are not as accurate.^58^ Designated experimental chambers or wind tunnels allowing plume shaping and visualization have been shown for flies, moths, cockroaches, bees and crabs.^53^, ^145^, ^146^, ^147^ Laboratory experiments facilitate the fine‐grained control of the odor environment,^35^, ^54^, ^148^ whilst simultaneously monitoring nearly all of the animal's behaviors. Such experiments with defined odor environments in *C. elegans a*nd *Drosophila* have given insight into the neural circuits underlying gradient navigation,^75^, ^149^ following volatile plumes^53^, ^60^, ^150^ and foraging in sparse food patches.^33^, ^151^, ^152^ Directly connecting the predictions of digestive and motor priming on foraging also requires quantifying the metabolic effects of odors. The genetic tractability of organisms such as worms or flies allows investigating signaling pathways, for example, to understand the interactions of digestive priming and internal states.

Recently, there were attempts to improve the lab environment by creating complex, scaffolded 3D structures to mimic outdoor habitats, in which worms navigate differently than on the artificially flat and uniform environment of a typical culturing plate.^34^ This study already identified novel behaviors like towering and jumping in *C. elegans*, which are likely to affect foraging by altering locomotion statistics and enabling the animals to sense volatile odors from a greater height. Video tracking can be adapted to a sufficient resolution to detect food intake and scale up existing algorithms for food intake detection.^153^, ^154^ Overall, invertebrate model systems can help fill the gaps in our understanding of foraging behavior, as food intake can be well monitored and accurate assays can be designed to reflect increasingly more realistic odors and resource distributions.

## 6 CONCLUSION

Foraging is a complex behavioral sequence that is highly dependent on nutritional needs, internal states and environmental resource distribution. Among the multiple sensory modalities that play a role in foraging, the smell is perhaps the most crucial. Odors carry information about the location and the quality of the food source. How this information is processed neuronally and the behavioral strategies that animals follow to select and locate nutrients have been well studied. Importantly, odor also primes the gastroenteric and feeding motor systems allowing animals to more efficiently use the resources available by, for example, increasing the effective rate of energy intake. Hence, odor is essential in the effective location and exploitation of food, although attention in the field has mostly been placed on the exploration portion. With the renewed appreciation for studying naturalistic behaviors,^155^ foraging is a key candidate for further theoretical and experimental studies as it is an ecologically meaningful, essential and animal‐initiated behavior.

We argue that a complete understanding of foraging can only be acquired by integrating knowledge across multiple disciplines and scales, from metabolomics to neuroscience. We suggest specifically that anticipatory feeding behaviors need to be included in foraging models, as they could have far‐reaching effects on the exploration–exploitation balance, besides their immediate effect on ingestion. Experimentally quantifying the role of odor sensing during foraging requires a faithful characterization of odor fields, locomotion and feeding and it is currently only feasible in laboratory settings using model organisms. Acquiring fine‐grained behavioral and metabolic data will be key in quantifying the impact of anticipatory feeding behaviors. Complementing these efforts, integrative models that combine sensory inputs and energetic considerations should be developed to bridge the gap between molecular descriptions and optimal foraging models. These efforts would make use of the deep insight existing separately in two distinct fields of neuroscience, namely the odor sensory and foraging communities.

7

**Glossary**
Biased random walks: random motion with a directional component
Continuous‐time random walks: a type of random walk with random waiting times between movements
Lévy flights: a type of random walk characterized by long runs
Drift‐diffusion model: a type of model where a variable that describes a decision (e.g., yes or no) changes based on the collected evidence. The resulting changes in the variable appear random, but with a directed component toward one option (see biased random walk)
Rheotaxis: motion in response to a water current
Chemotaxis: motion toward or away from a chemical
Anemotaxis: motion following airflow

## Data Availability

This is a review article and as such has no accompanying data.

## References

[1] Stöckl AL , Kelber A . Fuelling on the wing: sensory ecology of hawkmoth foraging. J Comp Physiol A Neuroethol Sens Neural Behav Physiol. 2019;205(3):399‐413. 30880349 10.1007/s00359-019-01328-2 PMC6579779

[2] Cardé RT . Multi‐cue integration: how female mosquitoes locate a human host. Curr Biol. 2015;25(18):R793‐R795. 26394099 10.1016/j.cub.2015.07.057

[3] Wittlinger M , Wehner R , Wolf H . The ant odometer: stepping on stilts and stumps. Science. 2006;312(5782):1965‐1967. 16809544 10.1126/science.1126912

[4] Wehner R , Lanfranconi B . What do the ants know about the rotation of the sky? Nature. 1981;293(5835):731‐733.

[5] Laidre ME . Foraging across ecosystems: diet diversity and social foraging spanning aquatic and terrestrial ecosystems by an invertebrate. Mar Ecol. 2013;34(1):80‐89.

[6] Hills TT , Todd PM , Lazer D , Redish AD , Couzin ID , Cognitive Search Research Group . Exploration versus exploitation in space, mind, and society. Trends Cogn Sci. 2015;19(1):46‐54. 25487706 10.1016/j.tics.2014.10.004 PMC4410143

[7] Baker KL , Dickinson M , Findley TM , et al. Algorithms for olfactory search across species. J Neurosci. 2018;38(44):9383‐9389. 30381430 10.1523/JNEUROSCI.1668-18.2018 PMC6209839

[8] Klein M , Krivov SV , Ferrer AJ , Luo L , Samuel AD , Karplus M . Exploratory search during directed navigation in *C. elegans* and *Drosophila* larva. eLife. 2017;6:e30503. doi:10.7554/eLife.30503 29083306 PMC5662291

[9] Gray JM , Hill JJ , Bargmann CI . A circuit for navigation in *Caenorhabditis elegans* . Proc Natl Acad Sci U S A. 2005;102(9):3184‐3191. 15689400 10.1073/pnas.0409009101 PMC546636

[10] Salvador LCM , Bartumeus F , Levin SA , Ryu WS . Mechanistic analysis of the search behaviour of *Caenorhabditis elegans* . J R Soc Interface. 2014;11(92):20131092. 24430127 10.1098/rsif.2013.1092 PMC3899880

[11] Buehlmann C , Mangan M , Graham P . Multimodal interactions in insect navigation. Anim Cogn. 2020;23(6):1129‐1141. 32323027 10.1007/s10071-020-01383-2 PMC7700066

[12] Kiss T . Do terrestrial gastropods use olfactory cues to locate and select food actively? Invert Neurosci. 2017;17(3):9. 28688004 10.1007/s10158-017-0202-2

[13] Kamio M , Derby CD . Finding food: how marine invertebrates use chemical cues to track and select food. Nat Prod Rep. 2017;34(5):514‐528. 28217773 10.1039/c6np00121a

[14] Lewis LPC , Siju KP , Aso Y , et al. A higher brain circuit for immediate integration of conflicting sensory information in *Drosophila* . Curr Biol. 2015;25(17):2203‐2214. 26299514 10.1016/j.cub.2015.07.015

[15] Vosshall LB , Stocker RF . Molecular architecture of smell and taste in *Drosophila* . Annu Rev Neurosci. 2007;30:505‐533. 17506643 10.1146/annurev.neuro.30.051606.094306

[16] Butterwick JA , Del Mármol J , Kim KH , et al. Cryo‐EM structure of the insect olfactory receptor Orco. Nature. 2018;560(7719):447‐452. 30111839 10.1038/s41586-018-0420-8 PMC6129982

[17] Hallem EA , Carlson JR . Coding of odors by a receptor repertoire. Cell. 2006;125(1):143‐160. 16615896 10.1016/j.cell.2006.01.050

[18] Horne JA , Langille C , McLin S , et al. A resource for the *Drosophila* antennal lobe provided by the connectome of glomerulus VA1v. eLife. 2018;7:e37550. doi:10.7554/eLife.37550 30382940 PMC6234030

[19] Bates AS , Schlegel P , Roberts RJV , et al. Complete connectomic reconstruction of olfactory projection neurons in the fly brain. Curr Biol. 2020;30(16):3183‐3199.e6. 32619485 10.1016/j.cub.2020.06.042 PMC7443706

[20] Mutlu AS , Gao SM , Zhang H , Wang MC . Olfactory specificity regulates lipid metabolism through neuroendocrine signaling in *Caenorhabditis elegans* . Nat Commun. 2020;11(1):1450. 32193370 10.1038/s41467-020-15296-8 PMC7081233

[21] Finger F , Ottens F , Springhorn A , et al. Olfaction regulates organismal proteostasis and longevity via microRNA‐dependent signaling. Nat Metab. 2019;1(3):350‐359. 31535080 10.1038/s42255-019-0033-z PMC6751085

[22] Smith KL , Baldwin RJ . Scavenging deep‐sea amphipods: effects of food odor on oxygen consumption and a proposed metabolic strategy. Mar Biol. 1982;68(3):287‐298.

[23] Cropper EC , Jing J , Perkins MH , Weiss KR . Use of the *Aplysia* feeding network to study repetition priming of an episodic behavior. J Neurophysiol. 2017;118(3):1861‐1870. 28679841 10.1152/jn.00373.2017 PMC5599670

[24] Susswein AJ , Kupfermann I , Weiss KR . The stimulus control of biting in *Aplysia* . J Comp Physiol. 1976;108(1):75‐96.

[25] Weiss KR , Chiel HJ , Koch U , Kupfermann I . Activity of an identified histaminergic neuron, and its possible role in arousal of feeding behavior in semi‐intact *Aplysia* . J Neurosci. 1986;6(8):2403‐2415. 3746414 10.1523/JNEUROSCI.06-08-02403.1986 PMC6568768

[26] Lum CS , Zhurov Y , Cropper EC , Weiss KR , Brezina V . Variability of swallowing performance in intact, freely feeding aplysia. J Neurophysiol. 2005;94(4):2427‐2446. 15944235 10.1152/jn.00280.2005 PMC1224712

[27] You Y‐J , Kim J , Cobb M , Avery L . Starvation activates MAP kinase through the muscarinic acetylcholine pathway in *Caenorhabditis elegans* pharynx. Cell Metab. 2006;3(4):237‐245. 16581001 10.1016/j.cmet.2006.02.012 PMC3433278

[28] Song B‐M , Faumont S , Lockery S , Avery L . Recognition of familiar food activates feeding via an endocrine serotonin signal in *Caenorhabditis elegans* . eLife. 2013;2:e00329. 23390589 10.7554/eLife.00329 PMC3564447

[29] Olsen SR , Wilson RI . Cracking neural circuits in a tiny brain: new approaches for understanding the neural circuitry of *Drosophila* . Trends Neurosci. 2008;31(10):512‐520. 18775572 10.1016/j.tins.2008.07.006 PMC2845908

[30] Dubnau J . Behavioral Genetics of the Fly (Drosophila Melanogaster). Cambridge University Press; 2014.

[31] Hammarlund M , Hobert O , Miller DM 3rd , Sestan N . The CeNGEN project: the complete gene expression map of an entire nervous system. Neuron. 2018;99(3):430‐433. 30092212 10.1016/j.neuron.2018.07.042 PMC6576255

[32] Harris TW , Arnaboldi V , Cain S , et al. WormBase: a modern model organism information resource. Nucleic Acids Res. 2020;48(D1):D762‐D767. 31642470 10.1093/nar/gkz920 PMC7145598

[33] Iwanir S , Brown AS , Nagy S , et al. Serotonin promotes exploitation in complex environments by accelerating decision‐making. BMC Biol. 2016;14:9. 26847342 10.1186/s12915-016-0232-y PMC4743430

[34] Guisnet A , Maitra M , Pradhan S , Hendricks M . A three‐dimensional habitat for *C. elegans* environmental enrichment. PLoS One. 2021;16(1):e0245139. 33428657 10.1371/journal.pone.0245139 PMC7799825

[35] Gershow M , Berck M , Mathew D , et al. Controlling airborne cues to study small animal navigation. Nat Methods. 2012;9(3):290‐296. 22245808 10.1038/nmeth.1853 PMC3513333

[36] Paulo DF , Junqueira ACM , Arp AP , et al. Disruption of the odorant coreceptor Orco impairs foraging and host finding behaviors in the New World screwworm fly. Sci Rep. 2021;11(1):11379. 34059738 10.1038/s41598-021-90649-x PMC8167109

[37] Asahina K , Pavlenkovich V , Vosshall LB . The survival advantage of olfaction in a competitive environment. Curr Biol. 2008;18(15):1153‐1155. 18674910 10.1016/j.cub.2008.06.075 PMC2575080

[38] Balkenius A , Rosén W , Kelber A . The relative importance of olfaction and vision in a diurnal and a nocturnal hawkmoth. J Comp Physiol A Neuroethol Sens Neural Behav Physiol. 2006;192(4):431‐437. 16380841 10.1007/s00359-005-0081-6

[39] Murlis J , Willis MA , Carde RT . Spatial and temporal structures of pheromone plumes in fields and forests. Physiol Entomol. 2000;25(3):211‐222.

[40] Murlis J , Jones CD . Fine‐scale structure of odour plumes in relation to insect orientation to distant pheromone and other attractant sources. Physiol Entomol. 1981;6(1):71‐86.

[41] Riffell JA , Shlizerman E , Sanders E , et al. Sensory biology. Flower discrimination by pollinators in a dynamic chemical environment. Science. 2014;344(6191):1515‐1518. 24970087 10.1126/science.1251041

[42] Weissburg MJ , Zimmer‐Faust RK . Odor plumes and how blue crabs use them in finding prey. J Exp Biol. 1994;197:349‐375. 7852909 10.1242/jeb.197.1.349

[43] Robinson EM , Smee DL , Trussell GC . Green crab (*Carcinus maenas*) foraging efficiency reduced by fast flows. PLoS One. 2011;6(6):e21025. 21687742 10.1371/journal.pone.0021025 PMC3110245

[44] Sprayberry JDH . The prevalence of olfactory‐ versus visual‐signal encounter by searching bumblebees. Sci Rep. 2018;8(1):14590. 30275496 10.1038/s41598-018-32897-y PMC6167322

[45] Manubay JA , Powell S . Detection of prey odours underpins dietary specialization in a neotropical top‐predator: how army ants find their ant prey. J Anim Ecol. 2020;89(5):1165‐1174. 32097493 10.1111/1365-2656.13188

[46] Pierce‐Shimomura JT , Morse TM , Lockery SR . The fundamental role of pirouettes in *Caenorhabditis elegans* chemotaxis. J Neurosci. 1999;19(21):9557‐9569. 10531458 10.1523/JNEUROSCI.19-21-09557.1999 PMC6782915

[47] Iino Y , Yoshida K . Parallel use of two behavioral mechanisms for chemotaxis in *Caenorhabditis elegans* . J Neurosci. 2009;29(17):5370‐5380. 19403805 10.1523/JNEUROSCI.3633-08.2009 PMC6665864

[48] Helms SJ , Rozemuller WM , Costa AC , Avery L , Stephens GJ , Shimizu TS . Modelling the ballistic‐to‐diffusive transition in nematode motility reveals variation in exploratory behaviour across species. J R Soc Interface. 2019;16(157):20190174. 31455164 10.1098/rsif.2019.0174 PMC6731512

[49] Moore P , Crimaldi J . Odor landscapes and animal behavior: tracking odor plumes in different physical worlds. J Mar Syst. 2004;49(1):55‐64.

[50] Vickers N . Mechanisms of animal navigation in odor plumes. Biol Bull. 2000;198(2):203‐212. 10786941 10.2307/1542524

[51] Wolf H , Wehner R . Pinpointing food sources: olfactory and anemotactic orientation in desert ants. Cataglyphis Fortis J Exp Biol. 2000;203(Pt 5):857‐868. 10667968 10.1242/jeb.203.5.857

[52] Buehlmann C , Graham P , Hansson BS , Knaden M . Desert ants locate food by combining high sensitivity to food odors with extensive crosswind runs. Curr Biol. 2014;24(9):960‐964. 24726153 10.1016/j.cub.2014.02.056

[53] Demir M , Kadakia N , Anderson HD , Clark DA , Emonet T . Walking *Drosophila* navigate complex plumes using stochastic decisions biased by the timing of odor encounters. eLife. 2020;9:e57524. doi:10.7554/eLife.57524 33140723 PMC7609052

[54] Albrecht DR , Bargmann CI . High‐content behavioral analysis of *Caenorhabditis elegans* in precise spatiotemporal chemical environments. Nat Methods. 2011;8(7):599‐605. 21666667 10.1038/nmeth.1630 PMC3152576

[55] Álvarez‐Salvado E , Licata AM , Connor EG , et al. Elementary sensory‐motor transformations underlying olfactory navigation in walking fruit‐flies. eLife. 2018;7:e37815. doi:10.7554/eLife.37815 30129438 PMC6103744

[56] Reidenbach MA , Koehl MAR . The spatial and temporal patterns of odors sampled by lobsters and crabs in a turbulent plume. J Exp Biol. 2011;214(Pt 18):3138‐3153. 21865526 10.1242/jeb.057547

[57] Li C , Dong H , Zhao K . A balance between aerodynamic and olfactory performance during flight in *Drosophila* . Nat Commun. 2018;9(1):3215. 30097572 10.1038/s41467-018-05708-1 PMC6086917

[58] Cardé RT , Willis MA . Navigational strategies used by insects to find distant, wind‐borne sources of odor. J Chem Ecol. 2008;34(7):854‐866. 18581182 10.1007/s10886-008-9484-5

[59] Vickers NJ . Winging it: moth flight behavior and responses of olfactory neurons are shaped by pheromone plume dynamics. Chem Senses. 2006;31(2):155‐166. 16339269 10.1093/chemse/bjj011

[60] van Breugel F , Dickinson MH . Plume‐tracking behavior of flying *Drosophila* emerges from a set of distinct sensory‐motor reflexes. Curr Biol. 2014;24(3):274‐286. 24440395 10.1016/j.cub.2013.12.023

[61] Singh J , Aballay A . Similar neural pathways control foraging in mosquitoes and worms. MBio. 2019;10(2):e00656–19. doi:10.1128/mBio.00656-19 31040241 PMC6495376

[62] Codling EA , Plank MJ , Benhamou S . Random walk models in biology. J R Soc Interface. 2008;5(25):813‐834. 18426776 10.1098/rsif.2008.0014 PMC2504494

[63] Sims DW , Humphries NE , Hu N , Medan V , Berni J . Optimal searching behaviour generated intrinsically by the central pattern generator for locomotion. eLife. 2019;8:e50316. doi:10.7554/eLife.50316 31674911 PMC6879304

[64] Maesani A , Ramdya P , Cruchet S , Gustafson K , Benton R , Floreano D . Fluctuation‐driven neural dynamics reproduce *Drosophila* locomotor patterns. PLoS Comput Biol. 2015;11(11):e1004577. 26600381 10.1371/journal.pcbi.1004577 PMC4657918

[65] Chevalier L , Coz M , Charrier M . Influence of inorganic compounds on food selection by the brown garden snail *Cornu aspersum (Müller) (Gastropoda: Pulmonata)* . Malacologia. 2003;45:125‐132.

[66] Chevalier L , Desbuquois C , Papineau J , Charrier M . Influence of the Quinolizidine alkaloid content of *Lupinus Albus (Fabaceae)* on the feeding choice of helix Aspersa *(Gastropoda: Pulmonata*). J Moll Stud. 2000;66:61‐68.

[67] Podroužková Š , Janovský Z , Horáčková J , Juřičková L . Do snails eat exotic plant species invading river floodplains? J Moll Stud. 2015;81(1):139‐146.

[68] Corrales‐Carvajal VM , Faisal AA , Ribeiro C . Internal states drive nutrient homeostasis by modulating exploration‐exploitation trade‐off. eLife. 2016;5:e19920. doi:10.7554/eLife.19920 27770569 PMC5108593

[69] Lindauer M . Über die Einwirkung von Duft‐ und Geschmacksstoffen sowie anderer Faktoren auf die Tänze der Bienen. Z Vgl Physiol. 1949;31(3):348‐412. 18131263

[70] Oberhauser FB , Czaczkes TJ . Tasting the unexpected: disconfirmation of expectations leads to lower perceived food value in an invertebrate. Biol Lett. 2018;14(9):20180440. 30185610 10.1098/rsbl.2018.0440 PMC6170749

[71] Abada EA‐E , Sung H , Dwivedi M , Park B‐J , Lee S‐K , Ahnn J . *C. elegans* behavior of preference choice on bacterial food. Mol Cells. 2009;28(3):209‐213. 19756391 10.1007/s10059-009-0124-x

[72] Katzen A , Chung H‐K , Harbaugh WT , et al. The nematode worm *C. elegans* chooses between bacterial foods exactly as if maximizing economic utility. Bio Rxiv; 2021. doi:10.1101/2021.04.25.441352 PMC10231927 37096663

[73] Worthy SE , Haynes L , Chambers M , et al. Identification of attractive odorants released by preferred bacterial food found in the natural habitats of *C. elegans* . PLoS One. 2018;13(7):e0201158. 30036396 10.1371/journal.pone.0201158 PMC6056031

[74] Li Z , Li Y , Yi Y , et al. Dissecting a central flip‐flop circuit that integrates contradictory sensory cues in *C. elegans* feeding regulation. Nat Commun. 2012;3(1):776. 22491324 10.1038/ncomms1780

[75] Larsch J , Flavell SW , Liu Q , Gordus A , Albrecht DR , Bargmann CI . A circuit for gradient climbing in *C. elegans* chemotaxis. Cell Rep. 2015;12(11):1748‐1760. 26365196 10.1016/j.celrep.2015.08.032 PMC5045890

[76] Harris G , Shen Y , Ha H , et al. Dissecting the signaling mechanisms underlying recognition and preference of food odors. J Neurosci. 2014;34(28):9389‐9403. 25009271 10.1523/JNEUROSCI.0012-14.2014 PMC4087214

[77] Chalasani SH , Chronis N , Tsunozaki M , et al. Dissecting a circuit for olfactory behaviour in *Caenorhabditis elegans* . Nature. 2007;450(7166):63‐70. 17972877 10.1038/nature06292

[78] Calhoun AJ , Chalasani SH , Sharpee TO . Maximally informative foraging by *Caenorhabditis elegans* . eLife. 2014;3:e04220. 25490069 10.7554/eLife.04220 PMC4358340

[79] Ben Arous J , Laffont S , Chatenay D . Molecular and sensory basis of a food related two‐state behavior in *C. elegans* . PLoS One. 2009;4(10):e7584. 19851507 10.1371/journal.pone.0007584 PMC2762077

[80] Sayin S , Boehm AC , Kobler JM , De Backer J‐F , Grunwald Kadow IC . Internal state dependent odor processing and perception‐the role of neuromodulation in the Fly olfactory system. Front Cell Neurosci. 2018;12:11. 29440990 10.3389/fncel.2018.00011 PMC5797598

[81] Münch D , Ezra‐Nevo G , Francisco AP , Tastekin I , Ribeiro C . Nutrient homeostasis ‐ translating internal states to behavior. Curr Opin Neurobiol. 2020;60:67‐75. 31816522 10.1016/j.conb.2019.10.004

[82] Vogt K , Zimmerman DM , Schlichting M , et al. Internal state configures olfactory behavior and early sensory processing in *Drosophila* larvae. Sci Adv. 2021;7(1):eabd6900. doi:10.1126/sciadv.abd6900 33523854 PMC7775770

[83] Kim SM , Su C‐Y , Wang JW . Neuromodulation of innate behaviors in *Drosophila* . Annu Rev Neurosci. 2017;40:327‐348. 28441115 10.1146/annurev-neuro-072116-031558

[84] Alcedo J , Prahlad V . Neuromodulators: an essential part of survival. J Neurogenet. 2020;34(3‐4):475‐481. 33170042 10.1080/01677063.2020.1839066 PMC7811185

[85] Ezcurra M , Walker DS , Beets I , Swoboda P , Schafer WR . Neuropeptidergic signaling and active feeding state inhibit nociception in *Caenorhabditis elegans* . J Neurosci. 2016;36(11):3157‐3169. 26985027 10.1523/JNEUROSCI.1128-15.2016 PMC4792932

[86] Root CM , Ko KI , Jafari A , Wang JW . Presynaptic facilitation by neuropeptide signaling mediates odor‐driven food search. Cell. 2011;145(1):133‐144. 21458672 10.1016/j.cell.2011.02.008 PMC3073827

[87] Sayin S , De Backer J‐F , Siju KP , et al. A neural circuit arbitrates between persistence and withdrawal in hungry *Drosophila* . Neuron. 2019;104(3):544‐558.e6. 31471123 10.1016/j.neuron.2019.07.028 PMC6839618

[88] Semmelhack JL , Wang JW . Select *Drosophila* glomeruli mediate innate olfactory attraction and aversion. Nature. 2009;459(7244):218‐223. 19396157 10.1038/nature07983 PMC2702439

[89] Ghosh DD , Sanders T , Hong S , et al. Neural architecture of hunger‐dependent multisensory decision making in *C. elegans* . Neuron. 2016;92(5):1049‐1062. 27866800 10.1016/j.neuron.2016.10.030 PMC5147516

[90] Lans H , Jansen G . Multiple sensory G proteins in the olfactory, gustatory and nociceptive neurons modulate longevity in *Caenorhabditis elegans* . Dev Biol. 2007;303(2):474‐482. 17187771 10.1016/j.ydbio.2006.11.028

[91] Alcedo J , Kenyon C . Regulation of *C. elegans* longevity by specific gustatory and olfactory neurons. Neuron. 2004;41(1):45‐55. 14715134 10.1016/s0896-6273(03)00816-x

[92] Libert S , Zwiener J , Chu X , Vanvoorhies W , Roman G , Pletcher SD . Regulation of *Drosophila* life span by olfaction and food‐derived odors. Science. 2007;315(5815):1133‐1137. 17272684 10.1126/science.1136610

[93] Zhang B , Jun H , Wu J , Liu J , Xu XZS . Olfactory perception of food abundance regulates dietary restriction‐mediated longevity via a brain‐to‐gut signal. Nat Aging. 2021;1(3):255‐268. 33796867 10.1038/s43587-021-00039-1 PMC8009090

[94] Sowa JN , Mutlu AS , Xia F , Wang MC . Olfaction modulates reproductive plasticity through neuroendocrine signaling in *Caenorhabditis elegans* . Curr Biol. 2015;25(17):2284‐2289. 26279229 10.1016/j.cub.2015.07.023 PMC4825799

[95] Díaz‐Muñoz M , Vázquez‐Martínez O , Aguilar‐Roblero R , Escobar C . Anticipatory changes in liver metabolism and entrainment of insulin, glucagon, and corticosterone in food‐restricted rats. Am J Physiol Regul Integr Comp Physiol. 2000;279(6):R2048‐R2056. 11080068 10.1152/ajpregu.2000.279.6.R2048

[96] Brandt C , Nolte H , Henschke S , et al. Food perception primes hepatic ER homeostasis via Melanocortin‐dependent control of mTOR activation. Cell. 2018;175(5):1321‐1335.e20. 30445039 10.1016/j.cell.2018.10.015 PMC6541012

[97] Glendinning JI , Lubitz GS , Shelling S . Taste of glucose elicits cephalic‐phase insulin release in mice. Physiol Behav. 2018;192:200‐205. 29621479 10.1016/j.physbeh.2018.04.002

[98] Boesveldt S , de Graaf K . The differential role of smell and taste for eating behavior. Perception. 2017;46(3‐4):307‐319. 28056650 10.1177/0301006616685576

[99] Lasschuijt MP , Mars M , de Graaf C , Smeets PAM . Endocrine cephalic phase responses to food cues: a systematic review. Adv Nutr. 2020;11(5):1364‐1383. 32516803 10.1093/advances/nmaa059 PMC7490153

[100] Fine LG , Riera CE . Sense of smell as the central driver of Pavlovian appetite behavior in mammals. Front Physiol. 2019;10:1151. 31620009 10.3389/fphys.2019.01151 PMC6759725

[101] Wang Y , Pu Y , Shen P . Neuropeptide‐gated perception of appetitive olfactory inputs in *Drosophila* larvae. Cell Rep. 2013;3(3):820‐830. 23453968 10.1016/j.celrep.2013.02.003

[102] Scholz M , Dinner AR , Levine E , Biron D . Stochastic feeding dynamics arise from the need for information and energy. Proc Natl Acad Sci. 2017;114(35):9261‐9266. 28802256 10.1073/pnas.1703958114 PMC5584422

[103] Stephens DW . Decision ecology: foraging and the ecology of animal decision making. Cogn Affect Behav Neurosci. 2008;8(4):475‐484. 19033242 10.3758/CABN.8.4.475

[104] Mobbs D , Trimmer PC , Blumstein DT , Dayan P . Foraging for foundations in decision neuroscience: insights from ethology. Nat Rev Neurosci. 2018;19(7):419‐427. 29752468 10.1038/s41583-018-0010-7 PMC6786488

[105] Nonacs P . State dependent behavior and the marginal value theorem. Behav Ecol. 2001;12(1):71‐83.

[106] Charnov EL . Optimal foraging, the marginal value theorem. Theor Popul Biol. 1976;9(2):129‐136. 1273796 10.1016/0040-5809(76)90040-x

[107] Arditi R , Dacorogna B . Optimal foraging on arbitrary food distributions and the definition of habitat patches. Am Nat. 1988;131(6):837‐846.

[108] Wajnberg E , Bernhard P , Hamelin F , Boivin G . Optimal patch time allocation for time‐limited foragers. Behav Ecol Sociobiol. 2006;60(1):1‐10.

[109] Trimmer PC , Houston AI , Marshall JAR , Mendl MT , Paul ES , McNamara JM . Decision‐making under uncertainty: biases and Bayesians. Anim Cogn. 2011;14(4):465‐476. 21360119 10.1007/s10071-011-0387-4

[110] Zoltowski D , Pillow J , Linderman S . A general recurrent state space framework for modeling neural dynamics during decision‐making. Proc Mach Learn Res. 2020;119:11680‐11691.

[111] Vergassola M , Villermaux E , Shraiman BI . “Infotaxis” as a strategy for searching without gradients. Nature. 2007;445(7126):406‐409. 17251974 10.1038/nature05464

[112] Davidson JD , Hady AE . Foraging as an evidence accumulation process. PLoS Comput Biol. 2019;15(7):e1007060. 31339878 10.1371/journal.pcbi.1007060 PMC6682163

[113] Iwanir S , Ruach R , Itskovits E , Pritz CO , Bokman E , Zaslaver A . Irrational behavior in *C. elegans* arises from asymmetric modulatory effects within single sensory neurons. Nat Commun. 2019;10(1):3202. 31324786 10.1038/s41467-019-11163-3 PMC6642097

[114] Cohen D , Teichman G , Volovich M , et al. Bounded rationality in *C. elegans* is explained by circuit‐specific normalization in chemosensory pathways. Nat Commun. 2019;10(1):3692. 31409788 10.1038/s41467-019-11715-7 PMC6692327

[115] Dweck HKM , Ebrahim SAM , Retzke T , et al. The olfactory logic behind fruit odor preferences in larval and adult *Drosophila* . Cell Rep. 2018;23(8):2524‐2531. 29791860 10.1016/j.celrep.2018.04.085

[116] Yoshida K , Hirotsu T , Tagawa T , et al. Odour concentration‐dependent olfactory preference change in *C. elegans* . Nat Commun. 2012;3:739. 22415830 10.1038/ncomms1750

[117] Choi JI , Yoon K‐H , Subbammal Kalichamy S , Yoon S‐S , Il LJ . A natural odor attraction between lactic acid bacteria and the nematode *Caenorhabditis elegans* . ISME J. 2016;10(3):558‐567. 26241504 10.1038/ismej.2015.134 PMC4817685

[118] Garlapati VK , Banerjee R . Solvent‐free synthesis of flavour esters through immobilized lipase mediated transesterification. Enzyme Res. 2013;2013:367410. 23819043 10.1155/2013/367410 PMC3683480

[119] Fink E , Louis M . Inter‐species comparison of the orientation algorithm directing larval chemotaxis in the genus *Drosophila*. bioRxiv; 2021. doi:10.1101/2021.09.17.460740

[120] Steck K , Veit D , Grandy R , et al. A high‐throughput behavioral paradigm for *Drosophila* olfaction ‐ the Flywalk. Sci Rep. 2012;2:361. 22511996 10.1038/srep00361 PMC3328172

[121] Adams TB , Cohen SM , Doull J , et al. The FEMA GRAS assessment of benzyl derivatives used as flavor ingredients. Food Chem Toxicol. 2005;43(8):1207‐1240. 15950815 10.1016/j.fct.2004.11.014

[122] McMeniman CJ , Corfas RA , Matthews BJ , Ritchie SA , Vosshall LB . Multimodal integration of carbon dioxide and other sensory cues drives mosquito attraction to humans. Cell. 2014;156(5):1060‐1071. 24581501 10.1016/j.cell.2013.12.044 PMC4007582

[123] Khalil AA , ur Rahman U , Khan MR , Sahar A , Mehmood T , Khan M . Essential oil eugenol: sources, extraction techniques and nutraceutical perspectives. RSC Adv. 2017;7(52):32669‐32681.

[124] Afify A , Potter CJ . Insect repellents mediate species‐specific olfactory behaviours in mosquitoes. Malar J. 2020;19(1):127. 32228701 10.1186/s12936-020-03206-8 PMC7106743

[125] Godley BJ , Blumenthal JM , Broderick AC , et al. Satellite tracking of sea turtles: where have we been and where do we go next? Endanger Species Res. 2008;4:3‐22.

[126] Osborne JL , Clark SJ , Morris RJ , et al. A landscape‐scale study of bumble bee foraging range and constancy, using harmonic radar. J Appl Ecol. 1999;36(4):519‐533.

[127] Woodgate JL , Makinson JC , Lim KS , Reynolds AM , Chittka L . Continuous radar tracking illustrates the development of multi‐destination routes of bumblebees. Sci Rep. 2017;7(1):17323. 29230062 10.1038/s41598-017-17553-1 PMC5725577

[128] Kays R , Crofoot MC , Jetz W , Wikelski M . ECOLOGY. Terrestrial animal tracking as an eye on life and planet. Science. 2015;348(6240):aaa2478. 26068858 10.1126/science.aaa2478

[129] Hussey NE , Kessel ST , Aarestrup K , et al. ECOLOGY. Aquatic animal telemetry: A panoramic window into the underwater world. Science. 2015;348(6240):1255642. 26068859 10.1126/science.1255642

[130] Bridge ES , Wilhelm J , Pandit MM , et al. An Arduino‐based RFID platform for animal research. Front Ecol Evol. 2019;7:257.

[131] Catarinucci L , Colella R , Mainetti L , et al. An animal tracking system for behavior analysis using radio frequency identification. Lab Anim. 2014;43(9):321‐327. 10.1038/laban.547 25141063

[132] Manoukis NC , Collier TC . Computer vision to enhance behavioral research on insects. Ann Entomol Soc Am. 2019;112(3):227‐235.

[133] Straw AD . Review of methods for animal videography using camera systems that automatically move to follow the animal. Integr Comp Biol. 2021;12:917‐925. doi:10.1093/icb/icab126 34117754

[134] Mathis MW , Mathis A . Deep learning tools for the measurement of animal behavior in neuroscience. Curr Opin Neurobiol. 2020;60:1‐11. 31791006 10.1016/j.conb.2019.10.008

[135] Walter T , Couzin ID . TRex, a fast multi‐animal tracking system with markerless identification, and 2D estimation of posture and visual fields. eLife. 2021;10:e64000. doi:10.7554/eLife.64000 33634789 PMC8096434

[136] Mathis A , Mamidanna P , Cury KM , et al. DeepLabCut: markerless pose estimation of user‐defined body parts with deep learning. Nat Neurosci. 2018;21(9):1281‐1289. 30127430 10.1038/s41593-018-0209-y

[137] Pereira TD , Aldarondo DE , Willmore L , et al. Fast animal pose estimation using deep neural networks. Nat Methods. 2019;16(1):117‐125. 30573820 10.1038/s41592-018-0234-5 PMC6899221

[138] Ratnayake MN , Dyer AG , Dorin A . Tracking individual honeybees among wildflower clusters with computer vision‐facilitated pollinator monitoring. PLoS One. 2021;16(2):e0239504. 33571210 10.1371/journal.pone.0239504 PMC7877608

[139] Imirzian N , Zhang Y , Kurze C , Loreto RG , Chen DZ , Hughes DP . Automated tracking and analysis of ant trajectories shows variation in forager exploration. Sci Rep. 2019;9(1):13246. 31519955 10.1038/s41598-019-49655-3 PMC6744467

[140] Risse B , Mangan M , Del Pero L , Webb B . Visual tracking of small animals in cluttered natural environments using a freely moving camera: Proceedings of the IEEE International Conference on Computer Vision Workshops. openaccess.thecvf.com; 2017:2840–2849.

[141] Liao Q , Cowen EA . The information content of a scalar plume – A plume tracing perspective. Environ Fluid Mech. 2002;2(1):9‐34.

[142] Connor EG , McHugh MK , Crimaldi JP . Quantification of airborne odor plumes using planar laser‐induced fluorescence. Exp Fluids. 2018;59(9):137.

[143] Boie SD , Connor EG , McHugh M , et al. Information‐theoretic analysis of realistic odor plumes: what cues are useful for determining location? PLoS Comput Biol. 2018;14(7):e1006275. 29990365 10.1371/journal.pcbi.1006275 PMC6054425

[144] Farrell JA , Murlis J , Long X , Li W , Cardé RT . Filament‐based atmospheric dispersion model to achieve short time‐scale structure of odor plumes. Environ Fluid Mech. 2002;2(1):143‐169.

[145] Denissenko P , Lukaschuk S , Breithaupt T . The flow generated by an active olfactory system of the red swamp crayfish. J Exp Biol. 2007;210(Pt 23):4083‐4091. 18025009 10.1242/jeb.008664

[146] Willis MA , Avondet JL , Zheng E . The role of vision in odor‐plume tracking by walking and flying insects. J Exp Biol. 2011;214(Pt 24):4121‐4132. 22116754 10.1242/jeb.036954 PMC3223117

[147] Ikeno H , Akamatsu T , Hasegawa Y , Ai H . Effect of olfactory stimulus on the flight course of a honeybee, *Apis mellifera*, in a wind tunnel. Insects. 2013;5(1):92‐104. 26462581 10.3390/insects5010092 PMC4592630

[148] Chronis N , Zimmer M , Bargmann CI . Microfluidics for in vivo imaging of neuronal and behavioral activity in *Caenorhabditis elegans* . Nat Methods. 2007;4(9):727‐731. 17704783 10.1038/nmeth1075

[149] Gomez‐Marin A , Louis M . Multilevel control of run orientation in *Drosophila* larval chemotaxis. Front Behav Neurosci. 2014;8:38. 24592220 10.3389/fnbeh.2014.00038 PMC3923145

[150] Gepner R , Mihovilovic Skanata M , Bernat NM , Kaplow M , Gershow M . Computations underlying *Drosophila* photo‐taxis, odor‐taxis, and multi‐sensory integration. eLife. 2015;4:e06229. doi:10.7554/eLife.06229 25945916 PMC4466338

[151] Calhoun AJ , Tong A , Pokala N , Fitzpatrick JAJ , Sharpee TO , Chalasani SH . Neural mechanisms for evaluating environmental variability in *Caenorhabditis elegans* . Neuron. 2015;86(2):428‐441. 25864633 10.1016/j.neuron.2015.03.026 PMC4409562

[152] Busch KE , Olofsson B . Should I stay or should I go? Worm. 2012;1(3):182‐186. 24058845 10.4161/worm.20464 PMC3670411

[153] Lee KS , Iwanir S , Kopito RB , et al. Serotonin‐dependent kinetics of feeding bursts underlie a graded response to food availability in *C. elegans* . Nat Commun. 2017;8(1):14221. 28145493 10.1038/ncomms14221 PMC5296638

[154] Scholz M , Lynch DJ , Lee KS , Levine E , Biron D . A scalable method for automatically measuring pharyngeal pumping in *C. elegans* . J Neurosci Methods. 2016;274:172‐178. 27474347 10.1016/j.jneumeth.2016.07.016

[155] Krakauer JW , Ghazanfar AA , Gomez‐Marin A , Mac Iver MA , Poeppel D . Neuroscience needs behavior: correcting a reductionist bias. Neuron. 2017;93(3):480‐490. 28182904 10.1016/j.neuron.2016.12.041

